# Integrated Kinetic and Probabilistic Modeling of the Growth Potential of Bacterial Populations

**DOI:** 10.1128/AEM.04018-14

**Published:** 2015-04-10

**Authors:** S. M. George, A. Métris, J. Baranyi

**Affiliations:** Institute of Food Research, Norwich Research Park, Norwich, United Kingdom

## Abstract

When bacteria are exposed to osmotic stress, some cells recover and grow, while others die or are unculturable. This leads to a viable count growth curve where the cell number decreases before the onset of the exponential growth phase. From such curves, it is impossible to estimate what proportion of the initial cells generates the growth because it leads to an ill-conditioned numerical problem. Here, we applied a combination of experimental and statistical methods, based on optical density measurements, to infer both the probability of growth and the maximum specific growth rate of the culture. We quantified the growth potential of a bacterial population as a quantity composed from the probability of growth and the “suitability” of the growing subpopulation to the new environment. We found that, for all three laboratory media studied, the probability of growth decreased while the “work to be done” by the growing subpopulation (defined as the negative logarithm of their suitability parameter) increased with NaCl concentration. The results suggest that the effect of medium on the probability of growth could be described by a simple shift parameter, a differential NaCl concentration that can be accounted for by the change in the medium composition. Finally, we highlighted the need for further understanding of the effect of the osmoprotectant glycine betaine on metabolism.

## INTRODUCTION

In food microbiology studies, the maximum specific growth rate of a bacterial population in a given static environment is routinely estimated by (mostly empirical) predictive models based on viable-count measurements (1) (www.combase.cc). These models take key environmental factors, such as temperature, pH, and water activity, as the explanatory variables. Their relative accuracy is between 5 and 10% if the prediction is made in the interpolation region of the data used to develop the model and if the effect of only one environmental factor is considered while the other factors are close to optimal (2).

The maximum specific growth rate, μ, is an autonomous parameter; i.e., it is independent of the history of the cells and depends only on the growth conditions. Compared to the growth rate, the lag period has been studied little, although its exact and quantitative characterization is vital to understand the ability of microbes to survive and proliferate under a wide range of conditions (3), and modeling the adaptation to environmental stress especially remains a challenge in food microbiology studies (4). Another parameter, which characterizes the adaptation of bacteria to environmental stress, is the probability of growth. It is usually modeled under static conditions, either independently of the growth rate (5, 6) or as part of a set of models describing the effect of environmental factors on various response parameters within a space where growth is possible (7–9). No attempt has yet been made to include probability of growth in kinetic models describing the temporal variation of bacterial cell concentration under stress conditions.

Under osmotic stress, bacteria divide into two subpopulations: one adapts and grows exponentially, while the other does not proliferate (10). This may be because the cells die or are in a persistent state (3) or are viable but nonculturable (10). In this case, the viable count growth curve exhibits a decrease after inoculation, described as the phoenix phenomenon (11). When trying to estimate the fraction of the initial cells, *p*_0_, that produces the exponentially growing lineage, the numerical task leads to an ill-conditioned problem (10).

It has been demonstrated that, theoretically, an approach based on a quantification of the physiological state of the growing cell population, defined by the expression α_0_ = exp(−μλ) (λ being the population lag time), is a quantity compatible with probability concepts (12). This is because α_0_, a number between 0 and 1, represents the fraction of the initial cell population, *x*_0_, that could have produced the observed exponential phase, had the cells grown from the α_0_*x*_0_ level at the specific rate μ without a lag period. This lends itself to introducing the growth potential of the population as follows.

The overall lag of the population, λ_*a*_, is defined as

(1) $$\lambda_{a}=-(\ln\;p_{0}+\ln\;\alpha_{0})/\mu$$

This parameter is a delay in the onset of exponential growth. The exponential growth phase can be described by the linear function

(2) $$y(t)=y(0)+\mu (t-\lambda_{a})$$

where *y*(*t*) is the natural logarithm of the cell concentration, meant for time *t* > λ_*a*_ (12). This concept was studied by K. Koutsoumanis (13), too, using colony counts.

We applied a method based on optical density measurements (14) for the parallel estimation of the main parameters above: the probability of growth, *p*_0_, the maximum specific growth rate, μ, and the physiological state parameter of the growing subpopulation. From these parameters, λ_*a*_ can be calculated. We utilized this method to study the effect of the medium composition on the probability of growth of Salmonella enterica serovar Typhimurium under osmotic stress.

## MATERIALS AND METHODS

### Strain.

Salmonella enterica serovar Typhimurium strain SL1344 was maintained in basic minimal medium (BMM) (10) with 40% glycerol or in tryptone soy broth (TSB) with 40% glycerol, stored at −80°C. Before each experiment, Salmonella was subcultured twice in BMM or TSB and incubated at 37°C for 7 h and 17 h.

### Growth conditions.

The osmotic stress of three media, Luria-Bertani medium (LB; 10 g liter^−1^ tryptone, 5 g liter^−1^ yeast extract, 10 g liter^−1^ NaCl), BMM, and BMM with glycine betaine (150 μM glycine betaine [Sigma Chemical Co., United Kingdom] added as an osmoprotectant), was increased by adding NaCl; LB with up to 55 g liter^−1^ NaCl added (equivalent to 6.5% total NaCl), BMM with up to 35 g liter^−1^ NaCl added, and BMM plus betaine with up to 55 g liter^−1^ NaCl added.

### Growth curves.

Cultures (17 h at 37°C) in TSB and BMM were diluted and inoculated into 100 ml of prewarmed (37°C) growth medium, from TSB to LB and from BMM to BMM both with and without glycine betaine. Sampling was carried out at appropriate intervals up to 15 days according to the growth conditions. The growth curves were measured by plate counts on tryptone soy agar (TSA) (CM0131; Oxoid). The experiments were repeated, and the results reported are averages for replicate samples.

### Bioscreen experiments.

Salmonella Typhimurium SL1344 was grown from frozen stock in BMM or TSB plus 40% glycerol for 7 h at 37°C. An inoculum of 25 μl was placed in 10 ml fresh BMM or TSB and incubated at 37°C for 17 h. Based on plate counts of test cultures in TSB or BMM incubated at 37°C for 17 h, a dilution series was devised to ultimately give a concentration of 1 cell in 50 μl. For the Bioscreen experiments, cultures were diluted in the test media, e.g., BMM plus NaCl and glycine betaine or LB plus NaCl, by serial 10-fold dilutions, and then these were further diluted 1 in 2 and, for the highest 10-fold dilution, up to 1 in 64. Wells of a pair of Bioscreen plates (100 wells on each plate) were then filled with 50 μl diluted cells and 350 μl test medium. Each well of the first plate was inoculated with a target concentration of 1 cell per well. This was obtained by a series of binary dilutions. On the other plate, 50 wells were inoculated with ca. 2 cells, 25 wells with 4 cells, 12 with 8 cells, and 5 with 16 cells. These cultures were suitable for studying the stochastic birth/death process of single (or at least few) cells. The remaining 8 wells of this second plate represented population kinetics: they were inoculated with ca 32, 64, 320, 640, 3,200, 6,400, 32,000, and 64,000 cells. Plates were incubated in the Bioscreen at 37°C for up to 10 days. The number of cells inoculated into each well was estimated from plate counts of the highest 10-fold dilution.

### Estimation of the probability of growth for a single cell (*p*_0_).

The total number of cells, ρ_*a*_, inoculated in the 100 wells of the first microtiter plate, was estimated by plate counts. The aim was to obtain ca. 1-cell/well via dilutions, so we made sure that ρ_*a*_ was around 100. The relative standard deviation (RSD) of the plate count method is usually ca. 10 to 15% (1). As shown in Table A1, our plate count accuracy was 10% or less, so we took RSD^2^[ρ_*a*_] to be ≈0.01.

After incubation in the Bioscreen, the number of negative wells (i.e., where no growth was detected) was recorded. Negative wells could appear only if the number of initial cells or the probability of growth was low. The number of cells in a well follows the Poisson distribution with the parameter ρ = ρ_*a*_/*n*. The expected total number of growing cells on the plate is $n\;\ln\;\frac{n}{w_{0}}\;$, where *w*_0_ is the number of negative wells (where no growth was observed). This formula is meaningful only for 0 < *w*_0_ < *n*.

An estimator for the probability of growth of a single cell is

(3) p0^=min (n ln nw0ρa,1)

The method of C. Dupont and J. C. Augustin (15) was based on the same formula. Note that, at our ρ_*a*_ ≈100-cell/plate target concentration, p0^<1 holds only if *w*_0_ > *n/e*, where *e* equals exp(1) ≈ 2.718. Therefore, we included only experiments where 37 < *w*_0_ < *n* = 100.

In Appendix, we provide a formula for the RSD^2^ of the numerator of p0^. Using the result that the RSD^2^ of the ratio of independent variables can be estimated by the sum of the RSD^2^ values of the numerator and denominator (16), the accuracy of the above p0^ statistic can be approximated by

(4) $$\text{RSD}\left({\hat{p}}_{0}\right)\approx \sqrt{0.01+\frac{\frac{1}{w_{0}}-\frac{1}{n}}{{\left(\text{ln }n-\ln w_{0}\right)}^{2}}}$$

**FIG 1:**
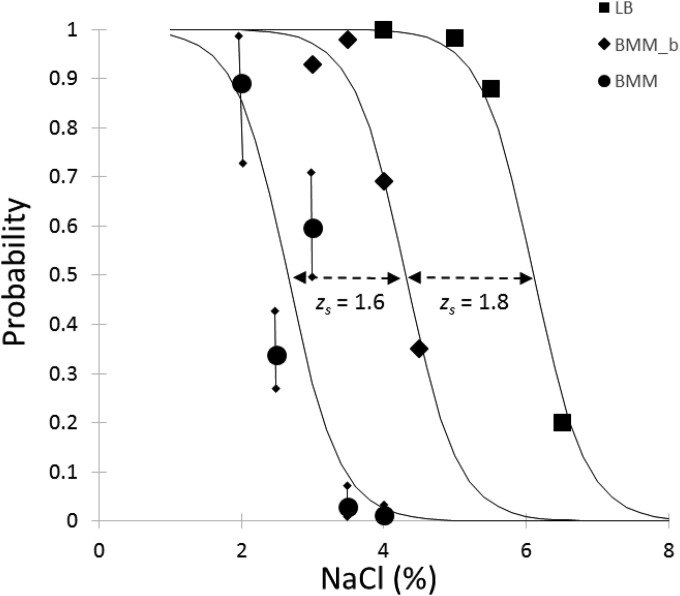
Effect of NaCl concentration on the probability of growth of Salmonella single cells. Data were obtained by Bioscreen based on equation 3. Error bars based on equation 4 are also shown for the results in BMM. The measured probabilities were fitted by weighted logistic regression. An *F* test showed that the maximum slopes could be considered identical in the three media; therefore, the *s*_0_ parameter (the NaCl percentage where the probability of growth is 0.5) characterizes the effect of the medium composition on the probability of growth: *s*_0_ = 6.1% in LB, 4.3% in BMM with glycine betaine (BMM_b), and 2.7% in BMM. The solid lines show the fit with the same maximum slope for the three media.

**FIG A1:**
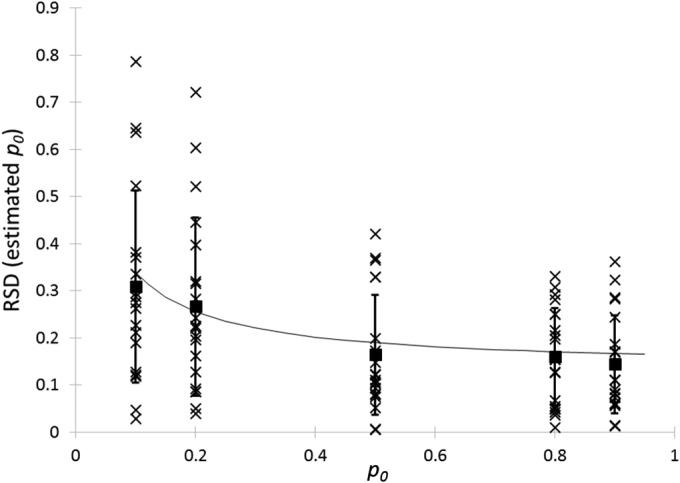
Simulation study where ρ_*a*_ (the number of cells on a plate of 100 wells) has an average value of 100 and its relative error is 10% (due to dilutions when the cells were inoculated into wells). The number of cells per well follows the Poisson distribution with the mean ρ value of ρ_*a*_/100. Each cell grows with *p*_0_ probability (*p*_0_ = 0.1, 0.2, 0.5, 0.8, and 0.9 on this plot). For each *p*_0_, ten p0^ independent estimates are shown (crosses) with their averages (squares with error bars). The theoretical estimate (line) is based on equation A6.

The error bars on Fig. 1 are calculated from the above estimation. A simulation study was also carried out to show that this is an accurate estimation of the expected relative error of p0^ (see Fig. A1).

### Estimation of the maximum specific growth rate, μ, and the physiological state, α_0_, from OD detection time data.

From an optical density (OD) curve of growth in a well, let *T*_det_ denote the time necessary to reach a detection level, which we set as an OD_det_ of 0.25. The *T*_det_ detection times were estimated for wells with high inocula. The maximum specific growth rate was estimated by the negative reciprocal of the differential quotient

(5) $$-\frac{1}{\mu }=\frac{{dT}_{\det\;}}{d\;\ln\;(\text{inoc})}$$

using only the last 8 wells where the expected number of inoculated cells was more than 30 (14). The physiological state of the growing subpopulation, α_0_, was determined by averaging the transformed *T*_det_ values of the other 192 wells, as in reference 14.

## RESULTS

### Primary model: growth response parameters obtained from Bioscreen experiments.

Table 1 shows the probability of growth for single cells of Salmonella, the physiological state, and the maximum specific growth rate of the growing subpopulation under various conditions in the three media. All three parameters decrease with NaCl concentration.

**TABLE 1:** Estimations of growth response parameters obtained from Bioscreen experiments at different NaCl concentrations in three different media

Medium	*s* (%)	*p*_0_^*a*^	α_0_^*b*^	ln μ (h^−1^)^*c*^
LB	4.0	1.00	0.0468	1.100
	5.0	0.98	0.0185	0.740
	5.5	0.88	0.0165	0.590
	6.5	0.20	0.0013	0.330
BMM + betaine	3.0	0.93	0.0418	0.474
	3.5	0.98	0.0365	0.384
	4.0	0.69	0.3208	0.350
	4.5	0.35	0.0187	0.270
BMM	2.0	0.89	0.0199	0.445
	2.5	0.34	0.0342	0.290
	3.0	0.60	0.0036	0.232
	3.5	0.03	ND^*d*^	0.200
	4.0	0.01	ND	0.140

a Physiological state of the growing subpopulation (17).

b Probability of growth.

c Natural log of the specific growth rate.

d ND, not determined. The physiological state could not be evaluated in these conditions because there were not enough data due to the low probability of growth.

### Secondary models: modeling the effect of NaCl and medium on the response parameters.

To compare the effect of the growth medium on *p*_0_, the data were fitted by weighted logistic regression as a function of *s*, the NaCl concentration, where the weights were derived from the above error estimation for p0^:

(6) p0^=(1+ea⋅[s−s0])−1

Here, *a* is a coefficient characterizing the maximum slope of the sigmoid curve describing the effect of NaCl variation on the probability of growth (Fig. 1), and *s*_0_ is the salt concentration where that probability is 0.5. An *F* test showed that the rate parameter *a* could be assumed to be the same in the different media (*P* = 0.56); therefore, it was a reasonable simplification that the three models for the probability of growth should differ from each other only by a *z_s_* shift in NaCl. The best fit for the parameter *a* was 2.7, while the *s*_0_ parameters were estimated as 6.1% in LB, 4.3% in BMM with glycine betaine, and 2.7% in BMM without osmoprotectant.

**FIG 2:**
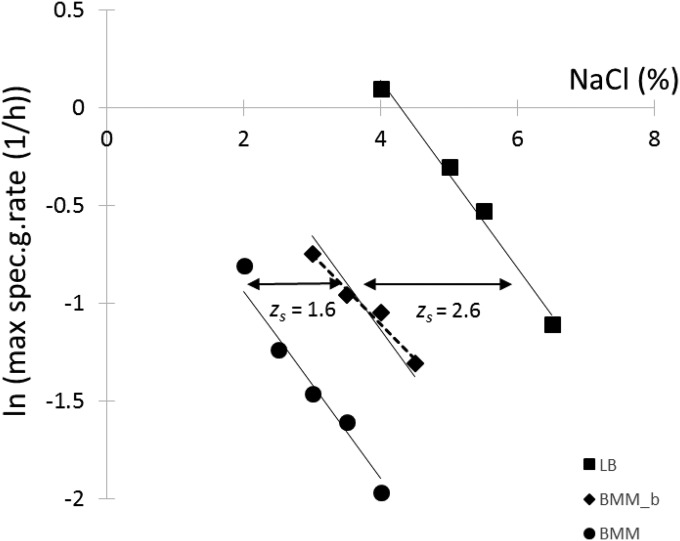
Effect of NaCl concentration on the natural logarithm of the specific growth rate (μ) of Salmonella. Data were obtained by Bioscreen based on equation 5. The measured maximum specific growth rates were fitted by linear regression. An *F* test showed that the slopes could be considered identical in the three media; therefore, the intercept with the NaCl axis, *s*_1_, characterizes the effect of the medium composition on the growth rate: *s*_1_ = 4.3% in LB, 1.6% in BMM with glycine betaine (BMM_b), and 0.04% in BMM. The broken line shows the fitted line if the measured ln(μ) values for BMM with glycine betaine had been fitted independently of the other data. The solid lines show the fit with the same slope for the three media.

A similar effect of the medium could be observed with the growth rate. Figure 2 shows the logarithm of the specific growth rates measured by Bioscreen as a function of NaCl in the three media. The data were fitted by linear regression. An *F* test showed that the three slopes could be considered identical (*P* = 0.12); therefore, only the intercept with the NaCl axis, *s*_1_, should depend on the medium. The common slope (*b*) of −0.478 and the intercept values of 4.3% in LB, 1.6% in BMM with glycine betaine, and 0.04% in BMM characterize the three responses. The shift in percent NaCl caused by the media is denoted by *z_s_* in the figures. Between BMM and BMM with glycine betaine, this shift turned out to be the same as with the model for the probability of growth.

**FIG 3:**
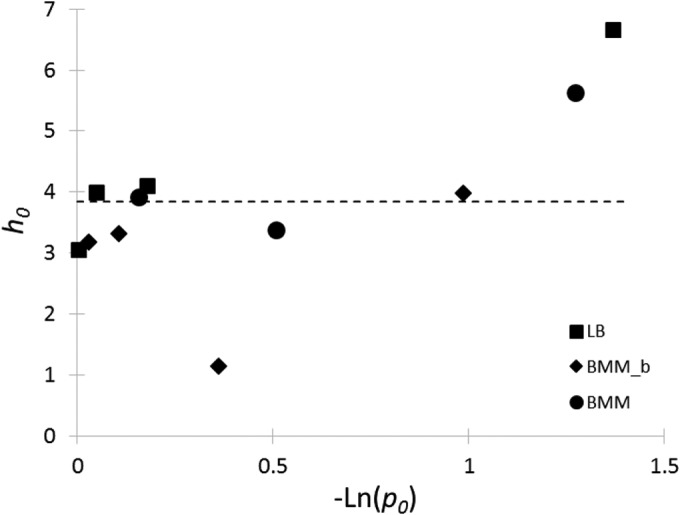
No strong correlation could be shown between the work-to-be-done parameter, *h*_0_ = −ln(α_0_), and the negative logarithm of the modeled probability of growth, −ln(*p*_0_), so *h*_0_ was taken as a constant, the average of its estimates, independently of the medium. BMM_b, BMM with glycine betaine.

Finally, the *h*_0_ = −ln(α_0_) values (17) were plotted against −ln(*p*_0_) as shown in Fig. 3. Only a slight positive correlation could be shown between them (*P* > 0.1), so *h*_0_ was taken as a constant, namely, the average of its estimates (3.85) from the primary models.

### Comparison of the response parameters obtained in the Bioscreen and by plate counts.

**FIG 4:**
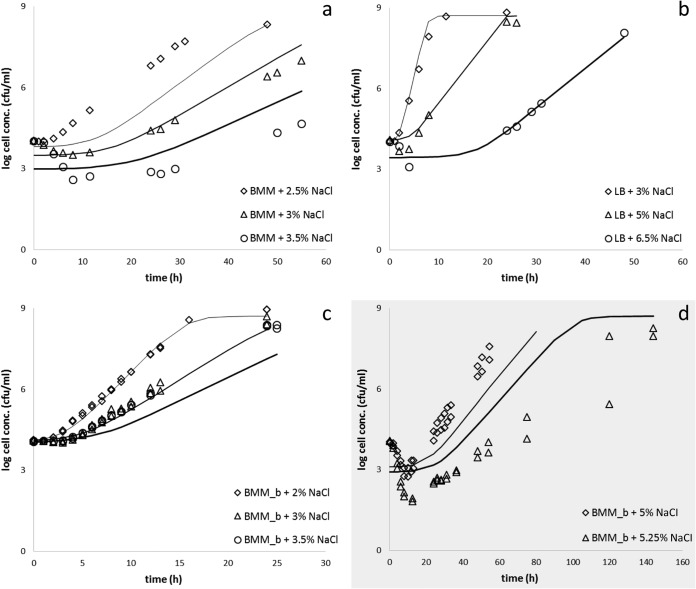
Comparison of the growth curves predicted from the Bioscreen data (continuous lines) with growth curves measured by plate counts in BMM (a), LB (b), and BMM with glycine betaine (BMM_b) (c) and extrapolation of the Bioscreen results in BMM with glycine betaine (d). The continuous lines represent the respective predicted curves of the growing subpopulation.

From the secondary models described above, growth curves were generated assuming that the growing subpopulation grows according to the model of Baranyi and Roberts (17) and that the nongrowing one follows linear death kinetics. The maximum population density was considered a constant, 5 × 10^8^ CFU ml^−1^ in all cases. The predictions, based on Bioscreen data, were compared to growth curves generated by plate counts. The results are shown in Fig. 4. The predictions were mostly in agreement with the plate count measurements except for the 2.5% NaCl concentration in BMM, where the lag time predicted was longer than that measured by plate count. The plate count was almost identical for 3 and 3.5% NaCl, though the Bioscreen results predicted ca. 20% less growth. At high salt concentrations, 5 and 5.25% NaCl in BMM with glycine betaine, we could not generate Bioscreen data, presumably due to the very small probability of growth. Therefore, the prediction at 5.25% NaCl is an extrapolation and, not surprisingly, differs from the data measured by plate counts (Fig. 4d).

## DISCUSSION

### Growth rate determined in the Bioscreen and by plate count.

**FIG 5:**
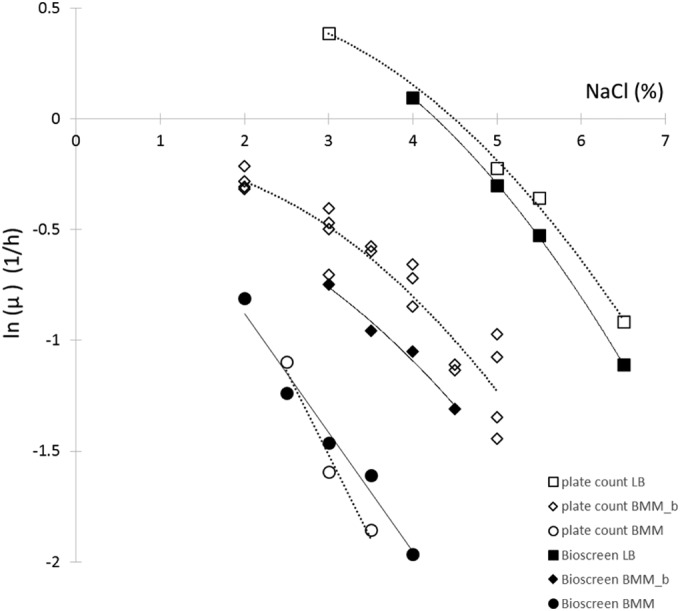
Comparison between the natural logarithm of the specific growth rates obtained from viable-count experiments and those from Bioscreen using different media. Glycine betaine is a less efficient osmoprotectant in the wells of the microtiter plate than in broth.

The measurement of bacterial kinetics by optical density has been widely studied. To estimate the specific growth rate by our method using Bioscreen data, an important condition is that the detection level be reached in the exponential phase and that the OD curves from different inocula be parallel. In this case, the method is robust, as shown by references 14, 18, and 19. Our validation data corroborate these findings; as shown in Fig. 5, there is no significant difference in the specific growth rate determined by viable counts and that determined by the Bioscreen, when the cells grow in BMM and in LB. However, with glycine betaine added to BMM, there is a systematic bias of more than 20%.

We observed the same difference (20) between the specific growth rate estimations, when Escherichia coli was grown under osmotic stress with and without glycine betaine or choline in the minimal medium. In a previous publication (20), we showed that the metabolic response of E. coli to osmotic stress in the presence of these osmoprotectants switched at a threshold NaCl concentration where the growth yield was optimum. With Salmonella, in the presence of glycine betaine, we found an optimum growth yield at the NaCl concentration where the probability of growth decreases (between 3.9 and 4.5% NaCl; data not shown).

The mechanism by which osmoprotectants work is not clear. Glycine betaine, for example, is believed to be metabolically inactive, but our results suggest that its interaction with metabolism may go beyond just restoring some of the turgor of the cell by replacing water (21, 22). Figure 5 suggests that the presence of glycine betaine in minimal medium had a protective effect compensating for a *z_s_* value of 1.6%, compared to minimal medium on its own in the Bioscreen, while it was about 2% under the same conditions when Salmonella was cultured in 100-ml vessels. Since the switch we observed with E. coli was from aerobic to fermentative pathways and the only parameter that may differ in the viable count and in the Bioscreen experiments is oxygen availability, it is reasonable to hypothesize that glycine betaine is also linked to respiration.

### The work-to-be-done parameter and osmotic stress.

As proven by (12), the observed overall lag time of the whole population (i.e., the delay to the onset of exponential growth) is

(7) $$\lambda_{a}=\lambda_{g}+\frac{-\ln\;(p_{0})}{\mu }$$

where *p*_0_ is the fraction of the initial cells whose lineages produce exponential growth, λ_*g*_ is the lag time of the growing subpopulation, and λ_*a*_ denotes the overall lag time (see also equations 1 and 2). S. J. Pirt (23) called λ_*a*_ “apparent lag.” Instead of replicate experiments, as described by Dupont and Augustin (15), the error of the p0^ statistic was estimated by equation 4, whose accuracy is shown in Fig. A1. It is an open question whether the overall variability shown in Fig. 1 can be largely explained by the measurement method or whether biological heterogeneity also significantly contributes to it.

The procedure elaborated in reference 14 was valid for an environment where all cells grew (*p*_0_ = 1), based on the mathematically proved theorem that, under certain homogeneity conditions, the average, α_0_, is independent of the inoculum. This parameter is convenient to quantify the physiological state, a kind of quantification of how suitable the growing cells are to the environment.

The expression β_0_ = α_0_ · *p*_0_ is a parameter quantifying the growth potential of the population. The natural logarithm of its first component, *h*_0_ = −ln(α_0_), has been used by several authors to quantify the “work to be done” by the growing cells. The second part takes into account that in fact only a fraction of the initial cell population is producing the exponential growth.

The *h*_0_ work-to-be-done parameter has been shown to be independent of the growth conditions if the cells are pretreated in a systematic way for stresses like temperature (24) but to increase with osmotic stress (10, 25). Figure 3 suggests that both *h*_0_ and −ln(*p*_0_) increase, so both α_0_ and *p*_0_ decrease with stress, but the correlation was weak (*P* > 0.1), and for the sake of simplicity, *h*_0_ was taken as a constant in the predictions. More data would be necessary for a decisive analysis.

### Effect of growth medium on the growth response parameters.

A quantification of bias is often used to compare the effect of the growth medium on the growth rate (8, 26). Here, we observed a systematic bias between the different media, although it depended on the measurement method; it was not the same when measured in the Bioscreen as when measured by plate count. A similar systematic shift could be observed for the probability of growth between the different media. In fact, this may be reformulated, based on a generalized *z* value concept, proposed by Pin et al. (27), as follows: in terms of probability of growth, the addition of glycine betaine to minimal medium is equivalent, at least in the studied region of NaCl, to a *z_s_* value of −1.6% NaCl, or a change from minimal medium to LB corresponds to a *z_s_* value of −3.5% NaCl. In terms of growth rate, the *z_s_* values as measured in the Bioscreen would be −1.6% and −4.2% NaCl, respectively. These concepts could help to develop useful interpretations for the food industry, where NaCl, a widely used preservative, needs to be reduced because of health issues.

### Conclusions.

In this study, we have shown that the introduced quantification of the growth potential of a bacterial culture, consisting of the proportion of the growing subpopulation (identified by the probability of growth for a single cell) and the suitability of these growing cells, is measurable by optical density experiments, in parallel with the specific growth rate of the population. These experiments lead to a robust description of the effect of rich and minimal media, with and without osmoprotectant, on the probability of growth of Salmonella under osmotic stress. A simple shift parameter of the added NaCl concentration answered the question of what amount of NaCl can be accounted for by adding an osmoprotectant. We also highlighted a gap in our biological understanding of the mechanism by which the osmoprotectant glycine betaine works, a typical area where systems biology approaches should be integrated in predictive modeling in food (28, 29).

It is important to see that, unlike the time to division for a single cell, the growth potential of an inoculated cell can be inferred retrospectively only, from the time when its isogenic descendants are growing exponentially. It can be envisaged as an environment- and history-dependent parameter encoded in the biochemical network of that initial single cell. The situation is similar to the specific growth rate that can be observed at the population level only; it is in fact a parameter derivable from the probability distribution of single-cell generation times.

The quantification, measurement, and modeling of growth potential of food-borne pathogens have special importance in quantitative microbial risk assessment, to which we hope to contribute with the present study.

## APPENDIX

It can be readily seen that the number of growing cells follow the Poisson distribution with the ρ*p*_0_ mean value, where ρ is the average number of initial cells in a well and *p*_0_ is the probability of growth for a cell.

The number of negative wells on a plate, *w*_0_, can be conceived as the number of failures in a Bernoulli experiment of *n* trials, with individual probabilities of *e^−ρp_0_^*. For the variance of their ratio,

(A1) $$\text{Var}\;[\frac{w_{0}}{n}]=\frac{e^{-{\rho p}_{0}}(1-e^{-\rho p_{0}})}{n}$$

From the first-order approximation of the natural log function around *e^−ρp_0_^*:

(A2) $$\text{Var}\;[\text{ln}\;\frac{n}{w_{0}}]=\text{Var}\;[\text{ln}\;\frac{w_{0}}{n}]\approx e^{2{\rho p}_{0}}\;\text{Var}\;[\frac{w_{0}}{n}]\approx \frac{e^{{\rho p}_{0}}-1}{n}\;$$

(A3) $${\text{RSD}}^{2}\;[\text{ln}\;\frac{w_{0}}{n}]\approx \frac{\left(e^{{\rho p}_{0}}-1)/({\rho p}_{0}\right)}{n{\rho p}_{0}}$$

Using ρ · *p*_0_ ≈ ln *n* − ln *w*_0_, a practical approximation can be derived:

(A4) $${\text{RSD}}^{2}\left[\ln\frac{w_{0}}{n}\right]\approx \frac{\frac{1}{w_{0}}-\frac{1}{n}}{{\left(\ln\;n-\ln\;w_{0}\right)}^{2}}$$

Table A1 shows that the accuracy of our plate count measurements is generally around or less than 10%; therefore, for our statistic

(A5) p0^=min (n ln nw0ρa, 1)

an accuracy estimation can be obtained as

(A6) $${\text{RSD}}^{2}\left[{\hat{p}}_{0}\right]\approx 0.01+\frac{\frac{1}{w_{0}}-\frac{1}{n}}{{\left(\ln n-\ln w_{0}\right)}^{2}}$$

This estimation was validated by a simulation study (Fig. A1).

**TABLE A1:** Three replicate plate counts to estimate cell concentration^*a*^

Medium	*s* (%)	Plate count (colonies/100 μl) in replicate			SD	RSD (%)
		1	2	3		
BMM	2	133	141	132	4.93	3.64
	2.5	119	135	138	10.21	7.82
	3	102	126	123	13.08	11.18
	3.5	129	116	147	15.57	11.91
BMM + betaine	3	131	137	143	6.00	4.38
	3.5	116	118	134	9.87	8.04
	4	135	122	139	8.89	6.73

a The relative error (RSD) is around or less than 10%. The RSD remains the same when the culture is diluted, so the estimated number of cells on a plate of 100 wells has the same relative error.
